# The thermostability of the RTS,S/AS01 malaria vaccine can be increased by co-lyophilizing RTS,S and AS01

**DOI:** 10.1186/s12936-020-03253-1

**Published:** 2020-06-08

**Authors:** Juliette Fortpied, Sylvie Collignon, Nicolas Moniotte, Frédéric Renaud, Babak Bayat, Dominique Lemoine

**Affiliations:** grid.425090.a GSK, Rue de l’Institut 89, 1330 Rixensart, Belgium

**Keywords:** AS01, Malaria, RTS,S, Thermostability, Vaccine

## Abstract

**Background:**

Developing thermostable vaccines is a challenge for pharmaceutical companies due to the inherent instability of biological molecules in aqueous solution. The problem is even more stringent in regions subjected to high temperatures in which protective cold chain is difficult to maintain due to a lack of infrastructure. Here, a simple, cost-effective solution to increase the thermostability of the malaria candidate vaccine RTS,S/AS01 is described. This vaccine currently needs to be stored between 2 and 8  °C due to the sensitivity of liquid AS01 to higher temperatures. The strategy was to increase thermostability by co-lyophilizing the RTS,S antigen and AS01.

**Methods:**

Co-lyophilization was achieved in a solution containing 5% sucrose, 10 mM potassium phosphate and 0.0312% polysorbate 80 at pH 6.1. The physicho-chemical characteristics and immunogenic properties of the resulting solid product, called CL-vac, fresh or stored at high temperature, were compared to those of the candidate RTS,S/AS01.

**Results:**

CL-vac proved to be acceptable in terms of visual appearance and physico-chemical characteristics. The structural integrity of both RTS,S and AS01 within CL-vac and its equivalence to the RTS,S/AS01 candidate vaccine were shown. Further, the stability of CL-vac was demonstrated for storage periods including 1 year at 4  °C, 1 year at 30  °C, and up to 6 months at 37  °C. In addition, CL-vac could withstand a heat excursion consisting of 1 month at 45  °C after storage for 1 year at 30  °C. Equivalence and stability were demonstrated by the various analytical tools and the immunogenicity of the samples after storage was also demonstrated in mice.

**Conclusions:**

In conclusion, the co-lyophilization process appeared as a promising approach to increase RTS/AS01 vaccine thermostability.

## Background

Today, the storage and distribution of a high number of vaccines require temperatures between 2 and 8  °C. Failure to respect such temperature constraint may damage antigen and/or adjuvant and reduce vaccine efficacy or safety [1]. Cold chain disruption can occur for several reasons, such as improperly maintained or outdated refrigeration equipment, loss of power to operate equipment, or poor compliance with cold chain procedures [2]. Despite improved efforts over the last decades to maintain cold chain conditions throughout vaccine transport, this constraint remains the most vulnerable part of immunization programmes [3]. Developing heat-stable vaccines that do not require refrigeration would have a major impact for global immunization programmes, by reducing deployment costs and vaccine wastage, thereby improving vaccine coverage [3, 4].

Vaccine instability is due to the inherent lability of biological molecules that are subject to a number of physical and chemical processes, i.e. unfolding, aggregation, hydrolysis or oxidation. A loss of vaccine potency may be the consequence. Most of these deleterious reactions occur in aqueous solution and are aggravated at higher temperatures, so removing water appears among the most efficacious ways for improving vaccine thermostability. However, drying of multi-component systems is more challenging than that of isolated molecules, due to the potential interactions between components [5, 6].

RTS,S/AS01 (GSK, Rixensart) is a malaria candidate vaccine that has recently completed a successful pivotal phase III safety, efficacy and immunogenicity study [7], and received a positive opinion from the European Medicines Agency [8]. This vaccine is presented as a two-vial liquid–solid formulation. One vial contains the lyophilized antigen (RTS,S) which has to be reconstituted with the content of the second vial, the Adjuvant System AS01 in liquid form. Lyophilized RTS,S is quite stable, but AS01 is temperature-sensitive, since its two immuno-enhancer components, 3-*O*-desacyl-4′-monophosphoryl lipid A (MPL) and *Quillaja saponaria* fraction 21 (QS-21) are subject to hydrolysis. As hydrolysis worsens with the temperature, AS01 should be stored refrigerated. Nevertheless, to simplify the storage procedure, the instructions are to keep both antigen and adjuvant between 2 and 8 °C.

The purpose of the present work was to develop a process leading to an RTS,S/AS01 malaria vaccine able to withstand higher temperatures than the refrigerated range. For cost-efficiency reasons, affordable technologies had to be used. Ensuring access and affordability of vaccines is of high importance in lower income country. Therefore, no change had to be made in the manufacturing process of both the antigen and adjuvant intermediate bulk, compared with the current candidate vaccine. The selected strategy was to use the same antigen and adjuvant and to co-lyophilize them in a single vial. The resulting solid form would need to be reconstituted in an aqueous vehicle just before administration. Whether the adjuvant would withstand the lyophilization process was unknown and represented one of the main challenges.

## Methods

### Vaccine components

The RTS,S antigen consists of a recombinant fusion protein (RTS) comprising the CS central tandem repeats and the C-terminal regions of the circumsporozoite protein (CSP) of *Plasmodium falciparum*, fused to the N-terminal region of the hepatitis B virus surface antigen (HBsAg). Co-expression of this fusion protein with native HBsAg in yeast results in the spontaneous formation of RTS,S virus-like particles (for review, see [9]).

The current RTS,S/AS01 candidate vaccine was used as control. It consists of a liquid AS01 adjuvant (25 µg QS-21 [licenced by GSK from Antigenics LLC, a wholly owned subsidiary of Agenus Inc, a Delaware, USA corporation] and 25 µg MPL [GSK] in a liposome-based formulation per 500 µl) and a lyophilized RTS,S batch (two-dose formulation). It will be referred to as the two-vial formulation throughout the text.

### Technical strategy

The selected development strategy was to prepare a new type of formulation, hereafter called co-lyophilized vaccine or CL-vac, by co-lyophilizing the antigen (RTS,S) together with the adjuvant (AS01). The formulation development was held with the aim to have a final product with a composition as close as possible to that of the two-vial formulation. Consequently, sucrose was selected as the cryo-protectant and potassium phosphate-pH 6.1 as the buffer. Phosphate was chosen because it is the buffer present in both the RTS,S and AS01 components of the two-vial formulation. The potassium form was selected because this is the most suitable for freeze-drying. The pH was set at 6.1, which is the pH of the AS01 formulation. It was preferred to the pH of the RTS,S formulation (which is 6.8) in order to increase AS01 stability, as the rate of MPL and QS-21 hydrolysis is lower at acidic pH than at basic pH. To obtain a formulation compatible with freeze-drying, the selected approach was to start from concentrated MPL-containing liposomes (1,2-dioleoyl-sn-phosphatidylcholine [DOPC] and cholesterol-based), to which concentrated QS-21 was added, and to let QS-21 quench to cholesterol before adding Polysorbate 80 and RTS,S.

### CL-vac preparation

First, sucrose was added to water for injection to a final concentration of 5% before addition of 10 mM potassium phosphate, pH 6.1. A solution of concentrated MPL-containing DOPC/cholesterol liposomes was added to reach a final concentration of 5 mg/ml DOPC, 1.25 mg/ml cholesterol and 0.25 mg/ml MPL. Next, QS-21 was added to reach a concentration of 0.25 mg/ml, and the resulting solution was stirred for 15–45 min at room temperature (throughout the text, room temperature is approximately 24  °C). Finally, 0.0312% (w/v) Polysorbate 80 and 0.25 mg/ml RTS,S were added. The formulation was stirred for 15–45 min at room temperature and the pH was measured and adjusted to 6.1 if needed. The solution was aliquoted into 3-ml siliconized vials (aliquot volume was 0.5 ml). Finally, the samples were lyophilized in a Martin Christ Epsilon lyophilizator with the same 28 h-freeze–drying cycle as that applied to the RTS,S antigen for the preparation of the two-vial formulation. More specifically, the vials were loaded onto shelves pre-chilled at − 52  °C and stayed at this temperature for 1 h under 1 Pa. The consecutive primary drying steps consisted of shelf temperature ramping from − 52  °C to − 13  °C in 2 h under 0.065 Pa, then − 13  °C during 6 h under 0.065 Pa, decreasing from − 13  °C to − 17  °C in 1 h under 0.065 Pa, and staying at −  17  °C for 8 h under 0.065 Pa. For the secondary drying step, the vials were submitted to temperature ramping from − 17  °C to +39  °C in 4 h under 0.05 Pa and stayed at +39  °C during 6 h under 0.05 Pa. After the secondary drying step, the chamber was backfilled with dry nitrogen up to a pressure of 825 mbar and stoppering occurred by collapsing the shelves of the freeze dryer.

All formulations were prepared in duplicate, starting from two different batches of purified RTS,S. When required for analysis, lyophilized samples were reconstituted in 150 mM NaCl.

### Stability

CL-vac was stored for up to 1 year at 4  °C or 30  °C, and for up to 6 months at 37  °C or 45  °C. In addition, after 1 year at 30  °C samples were stored for 1 month at 45  °C to simulate a more extreme heat excursion. Exposure at 45  °C was found relevant, as a malaria vaccine is intended for distribution in tropical areas. Samples taken at intermediate and final time points were kept at 4  °C until analysis.

### Electron microscopy (EM)

The samples were prepared for EM negative staining analysis according to a standard two-step negative staining method using sodium phosphotungstate as contrasting agent. A glow discharge was applied to the formvar/carbon-coated grid to enhance the adsorption of the sample. To better distribute the RTS,S particles and the AS, 200-mesh grids were used instead of 400-mesh grids for negative staining. Briefly, glow discharged carbon-formvar-coated nickel grids were floated on a 20 µl drop of the sample for 10 min at room temperature. Excess solution was blotted out. To remove most of the salts that may cause precipitation of the stain, the grid was briefly (30 s) floated on three drops of distilled water and then transferred on a drop of 2% (w/v) Na phosphotungstate in water, supplemented with 1% trehalose, which is blotted out after 30 s. The material was allowed to dry out thoroughly and was examined by transmission electron microscopy under a Zeiss Libra120 at 100 kV.

### Characterization of the antigen

The integrity of the RTS and S proteins within CL-vac was evaluated by sodium dodecyl sulfate–polyacrylamide gel electrophoresis (SDS-PAGE) under reducing and non-reducing conditions, by comparing with control RTS,S molecule. Analysis was performed following heating for 5 min at 95  °C in the presence of a standard Tris sample buffer (containing dithiothreitol as a reducing agent when needed) and run in Criterion XT 4–12% gels. Proteins were stained with Invitrogen Silver Xpress kit.

Size exclusion high performance liquid chromatography (HPLC-SEC) was selected to measure the aggregation status of the antigen in the presence of the adjuvant. An isocratic elution with 10 mM phosphate buffer (KH_2_/Na_2_H; pH 6.8), 25 mM NaCl and 0.05% Polysorbate-80 was applied at a flow-rate of 0.6 ml/min on a TSKGel G5000PWXL column (7.8 × 300 mm) with fluorescence detection (excitation at 280 nm and emission at 320 nm) to evaluate retention time and recovery.

RTS,S antigenicity was measured by enzyme-linked immunosorbent assay (ELISA) as follows: 96-well plates were coated overnight at 4  °C with a mouse anti-CSP monoclonal antibody at 1 µg/ml. After four washing steps with phosphate-buffered saline (PBS)-0.01% Tween 20 without calcium and magnesium, 200 µl of blocking buffer (PBS—1% casein) was added and the plates were incubated during 1 h at 37  °C. After washing, standard, internal control and RTS,S-containing samples were loaded, serially diluted in dilution buffer (PBS + 0.2% casein 0.2%, 0.1% tween) to reach a sample concentration estimated at 0.1 µg/ml and incubated for 2 h at 37  °C. After the plates have been washed, an HBsAg-specific rabbit polyclonal antibody was added (1/1000 in dilution buffer containing 0.5% mouse serum) and plates were incubated for 60 min. The next steps, with washings in between, were biotinylated donkey anti-rabbit Ig antibody (Amersham, N° RPN1004; 1/1000 in dilution buffer containing 0.5% mouse serum) for 1 h at 37  °C, and streptavidin–horseradish–peroxidase complex (Amersham, N° RPN1051; 1/1000 in dilution buffer containing 0.5% mouse serum) for 30 min at 37  °C. Eventually, the antibody complex was revealed by incubation with tetramethylbenzidine liquid substrate (TMB, Biorad 172-1072) for 15 min in the dark at room temperature and the reaction was stopped by addition of 100 µl H_2_SO_4_ 0.4 N. Optical density was recorded at 450–620 nm (Emax microplate reader, Molecular Devices, USA). Protein concentrations were determined using a four-parameter standard curve, back calculating against it for the controls and samples. For each sample the concentration was calculated relatively to the standard and was expressed in µg/ml.

### Characterization of the adjuvant

The size (Z-average diameter, ZAD, in nm) and polydispersity index (PDI) of adjuvant liposomes were measured in 96-well microplate by dynamic light scattering (DLS) on Dynapro II equipment (Wyatt Technology) against a polystyrene standard solution. Measurements of size and polydispersity were made in triplicate for each sample, also taking into account the viscosity of the samples. Results were expressed as the average of the three measurements.

QS-21 and hydrolyzed QS-21 (QS-21H) concentrations were determined by reversed-phase HPLC through a Symmetry RP18 column, with a water/acetonitrile gradient (in the presence of 0.05% trifluoroacetic acid) and UV detection at 214 nm.

The chemical integrity of MPL (percentage of MPL congeners) was determined following derivatization with dinitrobenzyloxyamine (DNBA) and reversed-phase HPLC through a Waters Symmetry C18 column and fluorescence detection (excitation at 345 nm and emission at 515 nm).

### Interaction between antigen and adjuvant

The reconstituted vaccine was submitted to ultracentrifugation in a sucrose gradient to separate RTS,S and AS01, followed by the quantification of RTS,S, DOPC and cholesterol (AS01 components) in the collected fractions. DOPC and cholesterol were quantified by a generic RP-HPLC method and the antigen by a specific RP-HPLC test. CL-vac samples reconstituted in 150 mM NaCl were compared with the two-vial formulation.

### Immunogenicity

The immunogenicity of CL-vac was compared to that of the two-vial formulation in CB6F1 mice. For immunization, 1/10th of human dose (2.5 µg RTS,S and 50 µl AS01) was injected intramuscularly on days 0, 14 and 28. CSP-specific and HBsAg-specific CD4^+^ T cell responses were evaluated 7 days after the third immunization. Anti-CSP and anti-HBsAg antibody responses were measured 14 days after the third immunization.

CSP and HBsAg-specific cellular responses were evaluated by intracellular cytokine staining, measuring the amounts of CSP- and HBsAg-specific CD4^+^ T cells expressing interferon (IFN)γ and/or IL-2 and/or tumor-necrosis factor (TNF), as already described [10]. Pools of 15-mers overlapping peptides covering either CSP or HBsAg were used for the stimulation.

Anti-CSP antibodies were measured using the same direct ELISA as the one developed for the two-vial formulation [11]. This ELISA is based on the binding to R32LR, which is a recombinant protein composed of major B cell epitopes of CSP [12, 13]. Anti-HBsAg antibodies were quantified as follows: 96-well plates were coated overnight at 4  °C with purified HBsAg 0.2 µg/ml. After washing with PBS-0.1% Tween-20 without calcium and magnesium, 200 µl blocking buffer (PBS—1% BSA—0.1% Tween 20—4% new born calf serum) was added and plates were incubated during 1 h at 37  °C. After washing with PBS-0.1% Tween-20, sera from immunized mice were serially diluted in blocking buffer and incubated in the plate for 1 h at 37  °C. Serial dilutions of the standard and control material were used to calculate anti-HBsAg antibody titers of the tested sera and to ensure validity of the test. Plates were washed with PBS-0.1% Tween-20 and horseradish peroxidase goat anti-mouse IgG (H+L) antibody (Jackson laboratory, No 115-035-003; 1/4000) was added for 1 h at 37  °C. After washing, the antibody complex was revealed by incubation with tetramethylbenzidine liquid substrate (TMB, Biorad 172-1072) for 15 min in the dark. The reaction was stopped by addition of 100 µl H_2_SO_4_ 0.4 N and optical density was recorded at 450–620 nm. The anti-HBsAg antibody titres of each individual mouse serum were determined from the standard curve using a regression model and the Soft Max Pro software. Geometric mean titers (GMT) were calculated for each group of mice.

### Animals and husbandry

Animal studies were ethically reviewed and carried out in accordance with European Directive 2010/63/EU and the GSK policy on the care, welfare and treatment of animals.

The CB6F1 mice were purchased from Harlan Laboratories B.V. (Horst, The Netherlands) and were aged 5 weeks upon arrival. Then, the animals were randomly distributed in different groups and were acclimated in polycarbonate cages type IV (Tecniplast, 1500 cm^2^) for a period of 9 days. The animals were randomly allocated to the study groups, with a maximum of ten mice per cage type III (Tecniplast, 800 cm^2^). All animals had free access to food (diet ref. A04-10 maintenance from SAFE) and 0.22-µm-filtered tap water. Nesting material was provided with nonstructural enrichment material (Envirodry). Bedding was made of sawdust Litaspen 8/20 (aspen which is heat-treated, dust-free and not treated by chemicals), and bedding change was performed every week. Certified bedding was purchased from a commercial supplier (Carfil).

Air supplied in housing room was 100% fresh air filtered by EPA filter and the ventilation was at least 20 cycles per hour. The animal room conditions were set as follows: temperature: 22  °C (± 2  °C); humidity: 55% (range from 40 to 65%) and light/dark cycle: 12 h/12 h. The pressure, temperature and relative humidity were recorded continuously by probes.

### Statistics

Fresh CL-vac and the two-vial formulation were compared by using an analysis of variance (ANOVA) on the log10 titers with group as fixed factor (identical variances were assumed). To study the stability of CL-vac, the sample size was chosen in order to demonstrate equivalence [0.5–2] for antibody responses and [0.33–3] for T-cell responses between the reference (two-vial formulation) and other stability conditions with at least 80% of power. The strategy was to successively demonstrate the equivalence between the reference and CL-vac stored for 6 months at 37  °C, the reference and CL-vac stored for 1 year at 4  °C, the reference and CL-vac stored for 1 year at 30  °C, and the equivalence between the reference and CL-vac stored for 1 year at 30  °C plus 1 month at 45  °C. The equivalence [0.5–2] for antibody and [0.33–3] for T-cell responses was defined as the geometric mean ratio between the processes and their 90% confidence interval within respectively [0.5–2] and [0.33–3]. The geometric mean ratios and their 90% confidence intervals were derived from an analysis of variance (ANOVA) model.

## Results

### Evaluation of extemporaneously reconstituted CL-vac

CL-vac and the two-vial formulation were obtained by two different processes, and the two vaccines had slightly different compositions (Table 1). Therefore, the equivalence of the two products had first to be demonstrated.

**Table 1:** Comparative composition of the RTS,S/AS01 two-vial formulation and colyophilized RTS,S/AS01 (CL-vac)

Vaccine components	Two-vial formulation	CL-vac
Sucrose (%)	2	1
PO_4_ (mM)	11	3
Liposome suspension (µg DOPC/mL)	1000	1000
Polysorbate 80 (%)	0.0062	0.0062
RTS,S antigen (µg/mL)	50	50
NaCl (mM)	150	153

Before reconstitution, it was observed that the cake appearance of CL-vac and that of RTS,S alone were similar, all showing an elegant pharmaceutical appearance. Residual moisture by µKarl Fischer was typically 0.3% for CL-vac and below the limit of quantification for RTS,S alone.

**Fig. 1:**
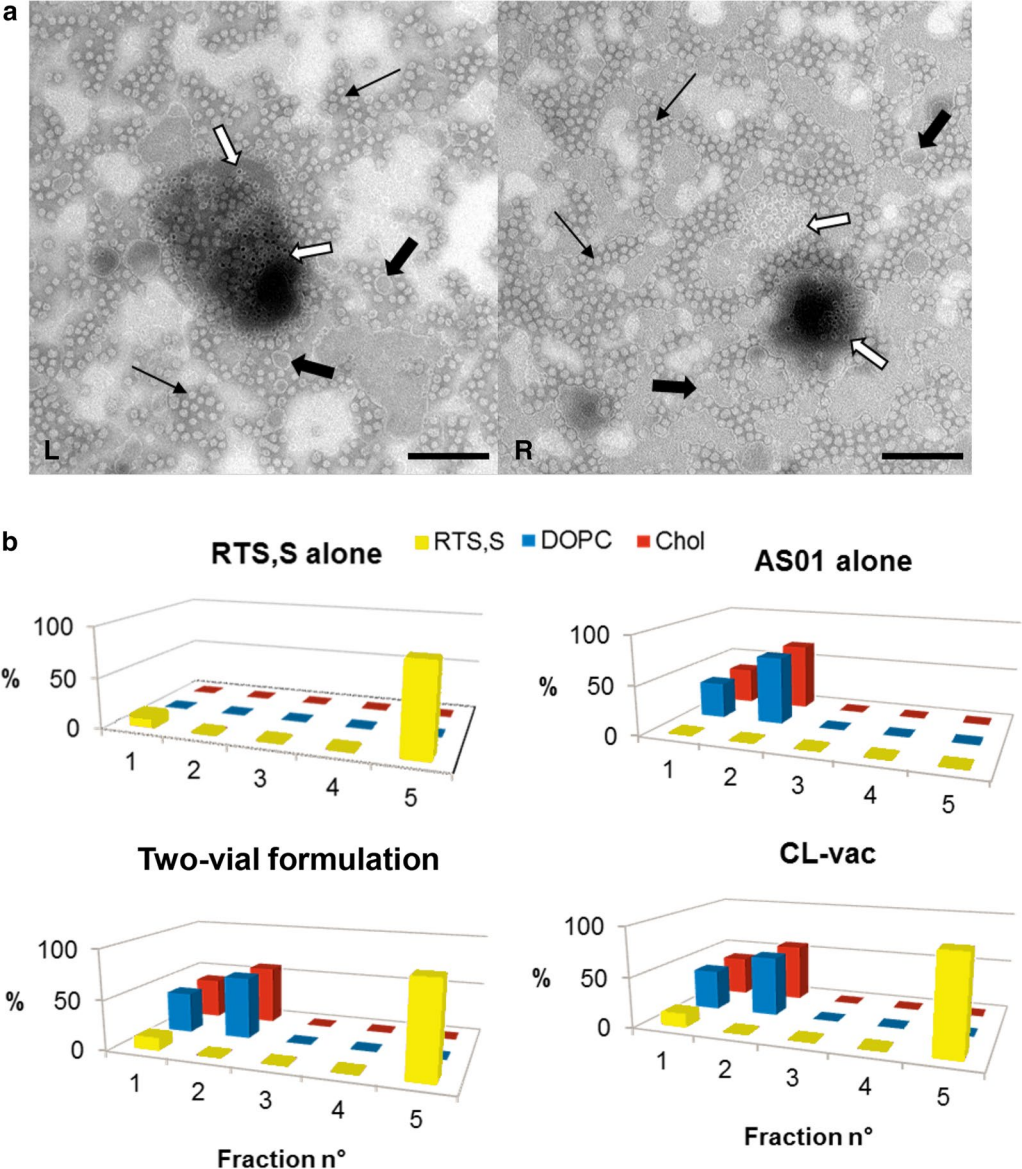
Evaluation of freshly reconstituted CL-vac. **a** Electronic microscopy analysis (TEM with negative staining) of reconstituted CL-vac (L) and reconstituted candidate RTS,S/AS01 two-vial formulation (R). White arrow, perforation of the liposome membrane. Thick black arrow, small liposome. Thin black arrow, RTS,S particles. Black bar represents 200 µm. **b** Interaction between antigen and adjuvant. RTS,S alone, AS01 alone, RTS,S/AS01 two-vial formulation (reconstituted vaccine) and CL-vac were centrifuged through a sucrose gradient. Five fractions were collected (fraction 1 at the top of the gradient, corresponding to low sucrose concentrations, and fraction 5 at the bottom). The concentration of RTS,S and DOPC and cholesterol (both AS01 components) were determined in the different fractions. The results showed no interaction between antigen in adjuvant in CL-vac, like in the RTS,S/AS01 two-vial formulation

When the two reconstituted products were examined by electron microscopy after negative staining, both showed well preserved and homogeneously spread RTS,S particles together with the typical structural pattern of AS01, including small liposomes and liposomes with perforated membranes (perforations of the liposome membranes are visible as small black dots corresponding to contrasting agent trapped in the holes/perforations) (Fig. 1a). The dark areas in Fig. 1a are commonly found in EM micrographs of our AS01 product. They are believed to result from an uneven distribution of AS01 adsorbed on the EM grid that can occur during sample preparation by negative staining. In regions where the density of the product is high, the contrasting agent can be trapped and accumulates, giving rise to these dark areas. Their presence and/or number is not a criterion for AS01 quality.

Comparison of the two samples by electron microscopy indicated that lyophilization did not impair the structural integrity of AS01 in CL-vac.

The two-vial formulation consists of a lyophilized RTS,S antigen to be reconstituted with AS01 in liquid form. Antigen and adjuvant do not interact with each other. We aimed to verify whether co-lyophilization impacted the interaction between the different components of the formulation in CL-vac (Fig. 1b). After separation on sucrose gradient and quantification of RTS,S, DOPC and cholesterol, adjuvant constituents and RTS,S were located in different fractions (DOPC and cholesterol separate in the lowest density region of the gradient; RTS,S was in the highest density fraction), both for CL-vac and for adjuvant and antigen loaded separately. If interaction took place between any of the constituents, the latter would be found together in the same fraction. The fact that all constituents of CL-vac were found in the same fractions as the individual components indicates that no interaction took place between RTS,S and AS01 in CL-vac.

**Fig. 2:**
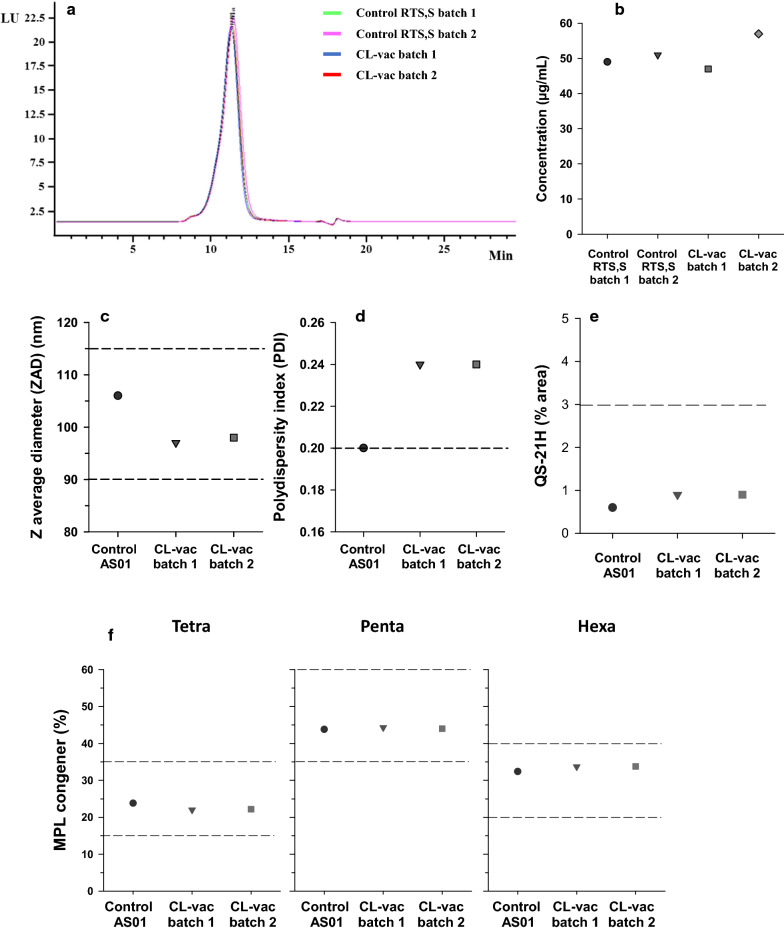
Evaluation of the antigen and adjuvant in freshly reconstituted CL-vac. **a**, **b** Analysis of two batches of CL-vac and two control RTS,S batches by SEC-HPLC and determination of RTS,S antigenicity as measured by ELISA. **c**, **d** The average liposome size (ZAD) and liposome size homogeneity (PDI) were determined for control AS01 and CL-vac (two batches). The dashed lines indicate the acceptance criteria set for the release of AS01 in liquid form (lower and upper limits for ZAD; upper limit for PDI). **e** The level of hydrolyzed QS-21 was measured in control AS01 and two batches of CL-vac. The dashed line indicates the upper threshold set for the release of AS01 in liquid form. **f** Determination of the proportion of MPL congeners (tetra, penta and hexa) in control AS01 and two batches of CL-vac. The dashed lines indicate the respective accepted ranges of congener proportions for the release of the adjuvant in liquid form

The quality attributes of each of the two vaccine components, antigen and adjuvant, were analysed. It was found that the size of the RTS,S particles, the antigen, was not altered by co-lyophilization, as indicated by their elution profile in SEC-HPLC (monitored by the retention time and relative abundance of the main peak) which was identical for RTS,S before lyophilization and RTS,S in CL-vac (Fig. 2a). The integrity of the individual RTS and S proteins in CL-vac was also confirmed by SDS-PAGE (results not shown). In addition, the antigenicity of the RTS,S particles in CL-vac was evaluated by ELISA and was found similar to that of control RTS,S (Fig. 2b). When analysing the adjuvant AS01 in CL-vac by DLS, it was observed that the size of its liposomes was slightly lower compared with the size observed in AS01 alone (approximately 97 nm versus 106 nm, respectively; Fig. 2c). Nevertheless, this difference was already present at the level of the final bulk (i.e. sample before lyophilization), indicating that this was not due to the lyophilization step. Further, the liposome size was slightly less homogeneous in CL-vac than in liquid AS01, as shown by the polydispersity index (Fig. 2d). Beyond the liposome characteristics, the chemical integrity of the AS01 immunoenhancers QS-21 and MPL was demonstrated in CL-vac, as the level of hydrolysed QS-21 (QS-21H) remained largely below 3%, which is the acceptance criterium set for the release of AS01 commercial batches (Fig. 2e), and the proportion of the different MPL congeners (hexa, penta and tetra) was the same as in control AS01 formulations and remained within the acceptance criteria set for AS01 release (Fig. 2f).

**Fig. 3:**
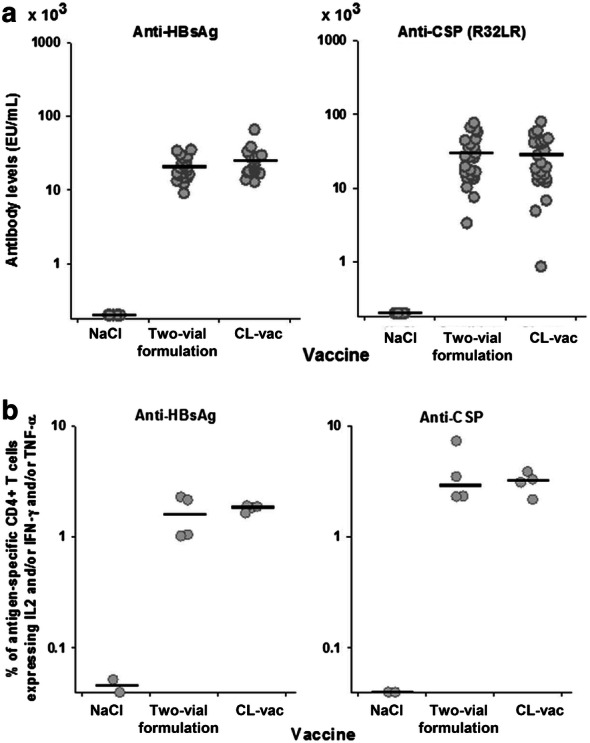
Evaluation of the immunogenic properties of CL-vac compared with the two-vial formulation. After reconstitution of the vaccines, mice were immunized three times intramuscularly and the induced specific antibody responses were measured 2 weeks after the third immunization by ELISA (anti-HBsAg and anti-R32LR) (**a**). The cellular responses (**b**) were evaluated on pooled blood 7 days after the third immunization by intracellular cytokine staining, measuring the percentage of CSP- and HBsAg-specific CD4 + T cells expressing IFNγ and/or IL-2 and/or TNFα. Immunization with the two-vial formulation was used as control. An investigational two-dose version of CL-vac was used in this experiment. One dot represents one mouse or one pool. The black bars are means for the antibody responses and medians for the cellular responses. The statistical analysis demonstrated the non-inferiority of CL-vac compared with the two-vial formulation, for all four measured parameters

The immunogenic properties of CL-vac were compared to that of the two-vial formulation. For that, mice were immunized with CL-vac or the two-vial formulation and the induced anti-CSP and anti-HBsAg cellular and humoral responses were evaluated (Fig. 3). For all four parameters, CL-vac was found equivalent to the two-vial formulation.

Altogether, these results indicated that the structural integrity of both antigen and adjuvant was similar in the two-vial formulation and CL-vac.

### Stability of CL-vac

Demonstrating the equivalence of CL-vac with the candidate RTS,S/AS01 was the essential first step. Further, the behaviour of the physico-chemical and immunogenic characteristics of CL-vac upon storage at different temperatures was investigated..

### Stability of the antigen

**Fig. 4:**
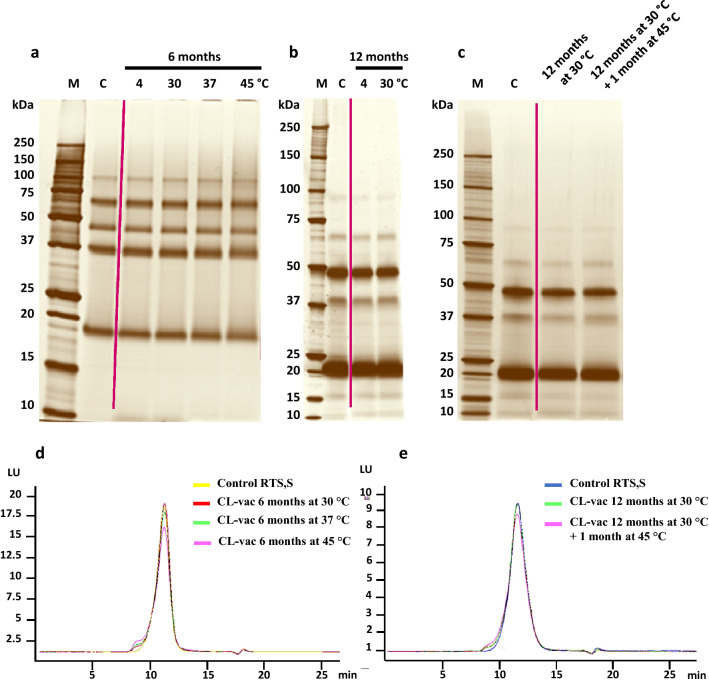
Evaluation of the RTS,S antigen in CL-vac after storage. **a–c** Silver-stained non-reducing SDS-PAGE gels showing the pattern of control RTS,S (lanes C) and RTS,S in CL-vac stored for 6 months at 4, 30, 37 or 45  °C, or 12 months at 4 or 30  °C, or 12 months at 30  °C + 1 month at 45  °C. Molecular mass markers (in kDa, lane M) are indicated on the left of each gel. **d**, **e** SEC-HPLC profiles of control RTS,S and RTS,S in CL-vac stored for 6 months at 30, 37 or 45  °C, or 12 months at 30  °C with or without an additional 1 month at 45  °C

The integrity of RTS,S in CL-vac reconstituted after storage was analysed by SDS-PAGE and SEC-HPLC. No major differences in the RTS,S profiles were observed between CL-vac stored for 6 months at 4, 30, 37 or 45  °C (Fig. 4a). Only some more smear was visible in the RTS,S profile of CL-vac stored at 45  °C. CL-vac was also stored for up to 1 year at 4 or 30  °C. Again, the corresponding RTS,S profiles in SDS-PAGE did not differ from that of a control RTS,S (Fig. 4b). Finally, we could see that 1 year at 30  °C followed by a heat excursion at 45  °C for 1 month did not affect the SDS-PAGE profile of CL-vac RTS,S (Fig. 4c).

Likewise, no differences in the SEC-HPLC elution patterns of RTS,S were observed upon storage of CL-vac for 6 months at 30 or 37  °C. However, a slight shoulder eluting ahead of the main peak was present in CL-vac samples but not in control RTS,S, which was more visible after 6 months at 45 °C. This may correspond to some slight aggregation of RTS,S particles, relating to the smear observed in SDS-PAGE (Fig. 4d). Twelve months at 30  °C with or without heat excursion did not impact the size of the RTS,S particles either, as could be judged by their elution profile in SEC-HPLC (Fig. 4e).

**Fig. 5:**
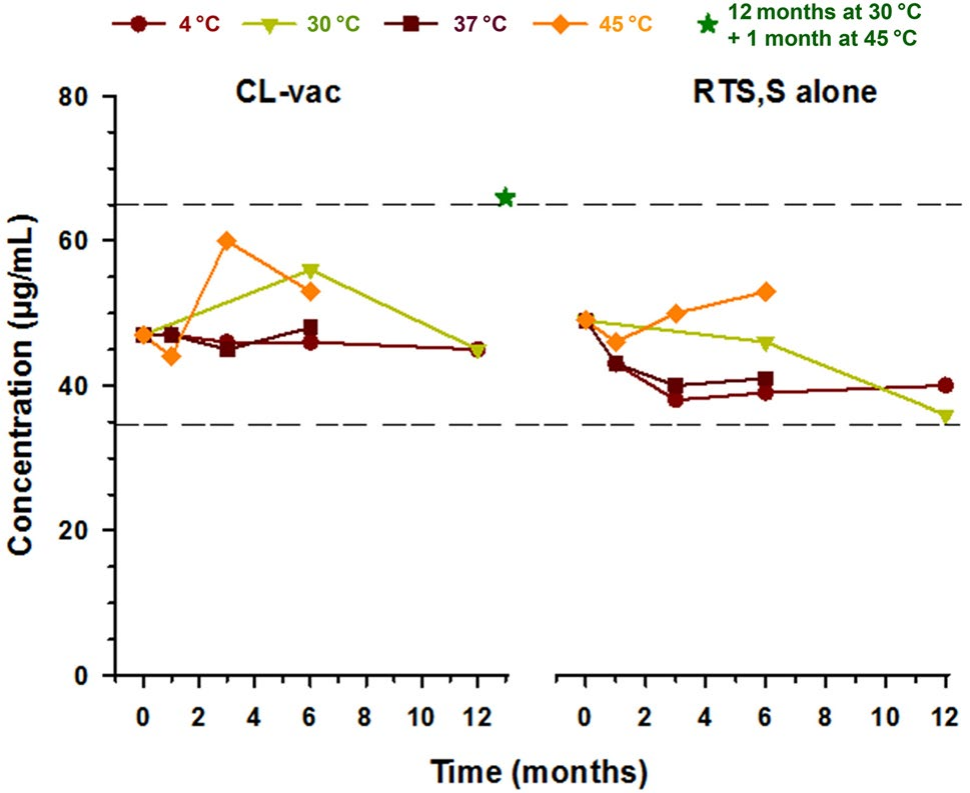
Evolution of RTS,S antigenicity upon storage. Isolated RTS,S and CL-vac were stored at 4, 30, 37 or 45  °C for up to 12 months and RTS,S antigenicity was measured by ELISA at different time points. In addition CL-vac was stored for 12 months at 30  °C followed by 1 month at 45  °C and RTS,S antigenicity was measured at this final time point. The dashed lines indicate the acceptance criteria set for RTS,S release

RTS,S antigenicity was not much affected either by storage at 4, 30, 37 or 45  °C (Fig. 5. Nevertheless, an increase in antigenicity, just above the acceptance criteria currently defined for RTS,S, was noticed after 1 year at 30  °C followed by the heat excursion at 45  °C.

### Stability of the adjuvant

The different components of the adjuvant, i.e. liposomes, QS-21 and MPL, were analysed independently.

**Fig. 6:**
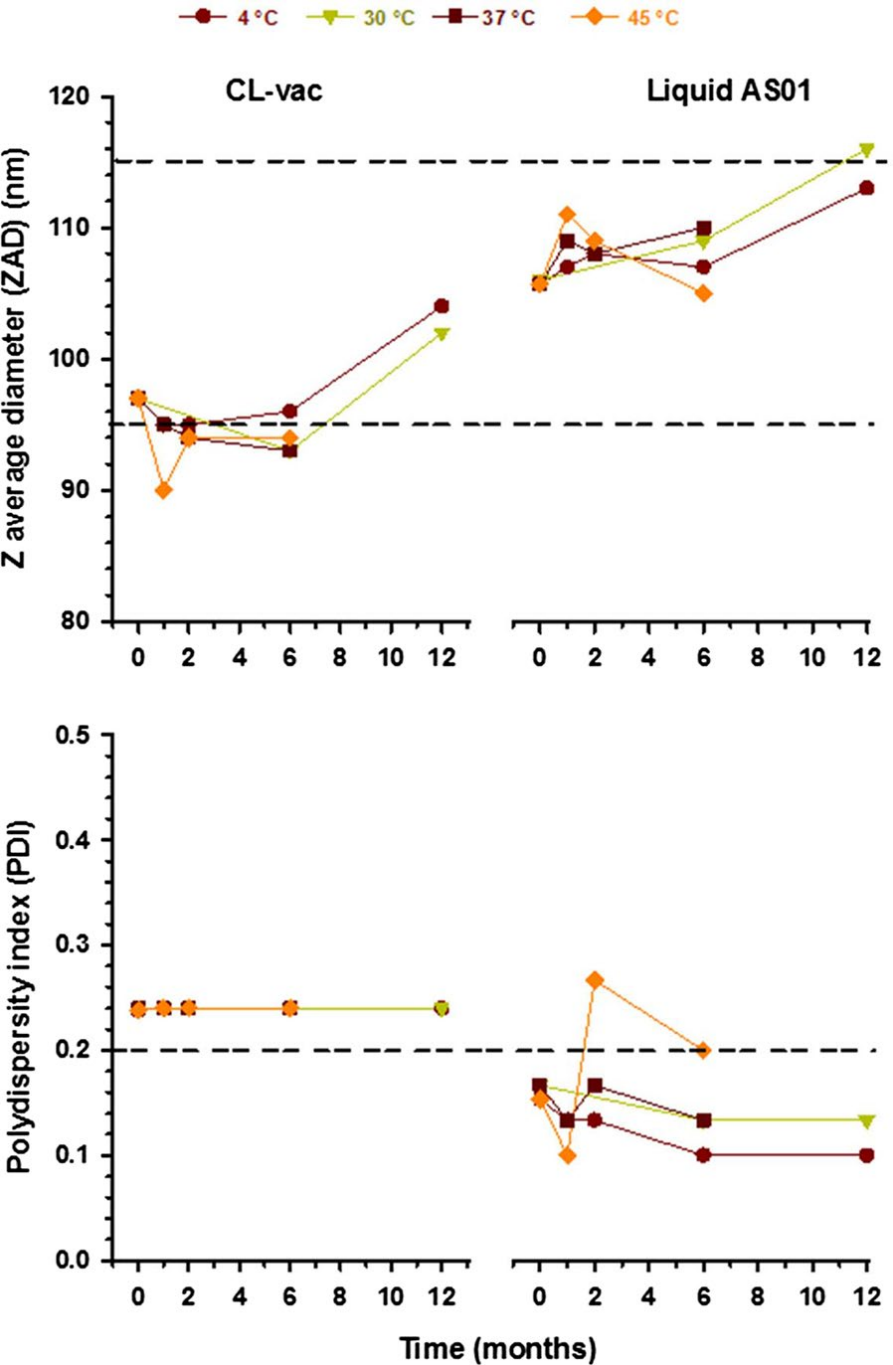
Evolution of the Z-average diameter (ZAD) and polydispersity index (PDI) measured by DLS upon storage. Liquid AS01 and CL-vac were stored at 4, 30, 37 or 45  °C for up to 12 months and (ZAD) and (PDI) were measured at different time points. The dashed lines indicate the acceptance criteria set for liquid AS01 release (lower and upper limits are shown for ZAD and upper limit is shown for PDI)

When evaluating extemporaneously reconstituted CL-vac at an earlier stage, we noticed that the size of the liposomes was smaller compared with that of the liposomes in AS01, reaching the lower limit of the criteria set for the release of liquid AS01. Upon storage at 4 and 30  °C, the size of the liposomes in CL-vac increased slightly, but the same observation was made in liquid AS01 so that the liposomes in CL-vac eventually remained smaller than the liposomes in liquid AS01 (Fig. 6).

A slightly higher polydispersity index (PDI) was observed in CL-vac compared with liquid AS01 (0.24 vs 0.20), which means more variation in liposome size in freshly reconstituted CL-vac than in AS01 (Fig. 6). However, this difference was not considered relevant and could be explained by a different matrix (RTS,S, sucrose and polysorbate-80 are present in CL-vac whereas these are not present in liquid AS01). The PDI remained stable in CL-vac upon storage independently of the temperature. For liquid AS01, a slight variation in PDI was observed at 45  °C (ranging from 0.18 to 0.23).

**Fig. 7:**
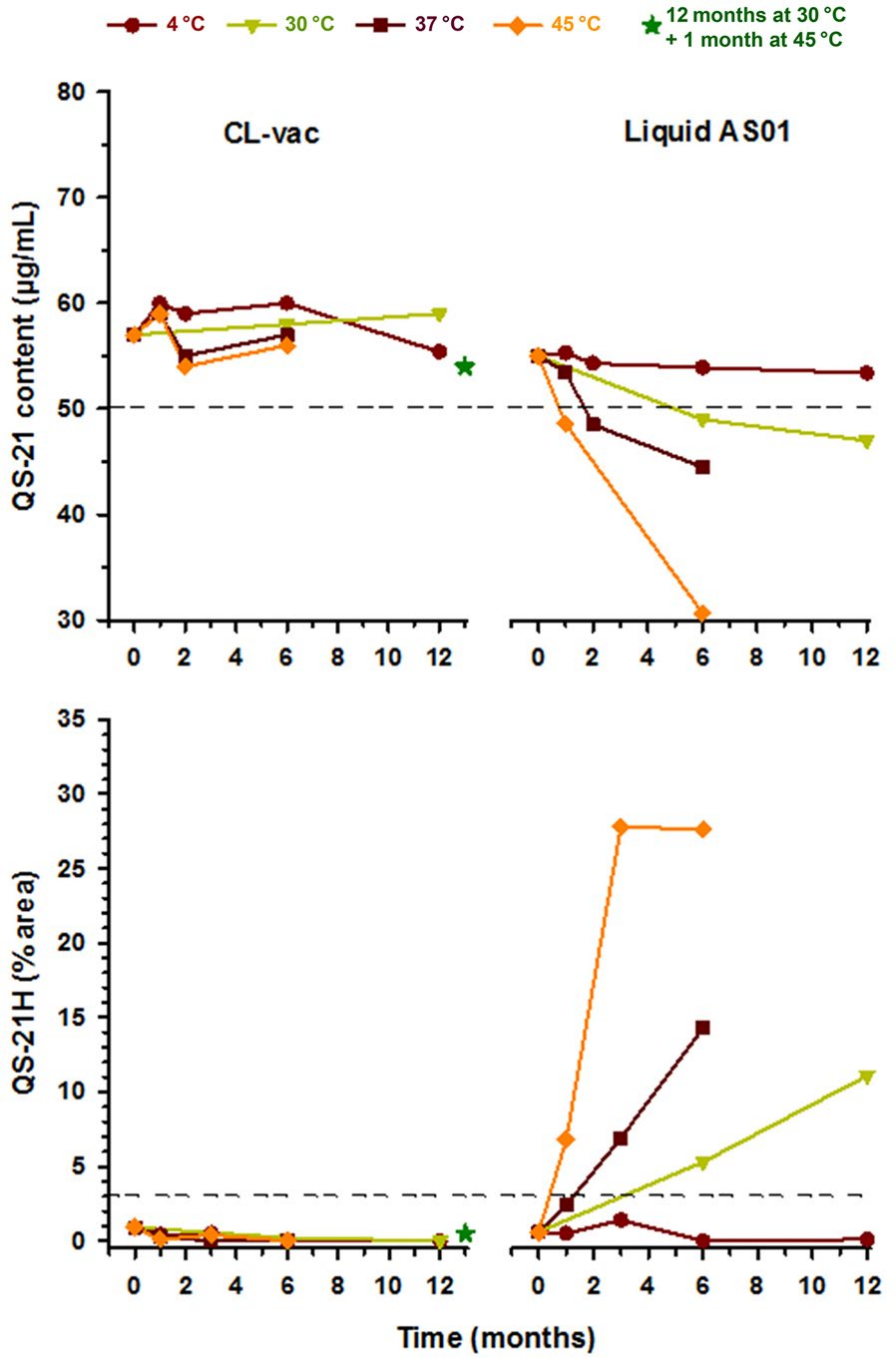
Evolution of QS-21 content and QS-21 hydrolysis upon storage. Liquid AS01 and CL-vac were stored at 4, 30, 37 or 45  °C for up to 12 months and the QS-21 content and the level of QS-21 hydrolysis were measured at different time points. A short heat expedition was made for CL-vac consisting of 1 month at 45  °C after storage for 12 months at 30  °C and the two parameters were also measured at this final time point. The dashed lines indicate the respective acceptance criteria set for the release of liquid AS01 (lower limit for QS-21 content and upper limit for QS-21H). Black bar represents 200 µm

Concerning QS-21, we could see that it was stable in CL-vac in all conditions of temperature and storage conditions, as no hydrolysis occurred. In contrast, in liquid AS01, QS-21 hydrolysis was temperature-dependent, and at 45  °C only 1 month of storage was needed to exceed the 3% level. Three months were needed at 37  °C and 6 months at 30  °C. The level of hydrolyzed QS-21 in liquid AS01 remained approximately the same at 4  °C, below 3% (Fig. 7). Consequently, QS-21 content in CL-vac stayed beyond 50 µg/ml in all conditions of temperature and storage time, whereas this parameter was stable in liquid AS01 only when storage occurred at 4  °C. At 30  °C, QS-21 content fell under the 50 µg/ml limit after 6 months of storage, reflecting QS21 hydrolysis. It only took 2 months or even 1 month at 37  °C or 45  °C, respectively, to see the QS-21 content under 50 µg/ml in liquid AS01 (Fig. 7).

**Fig. 8:**
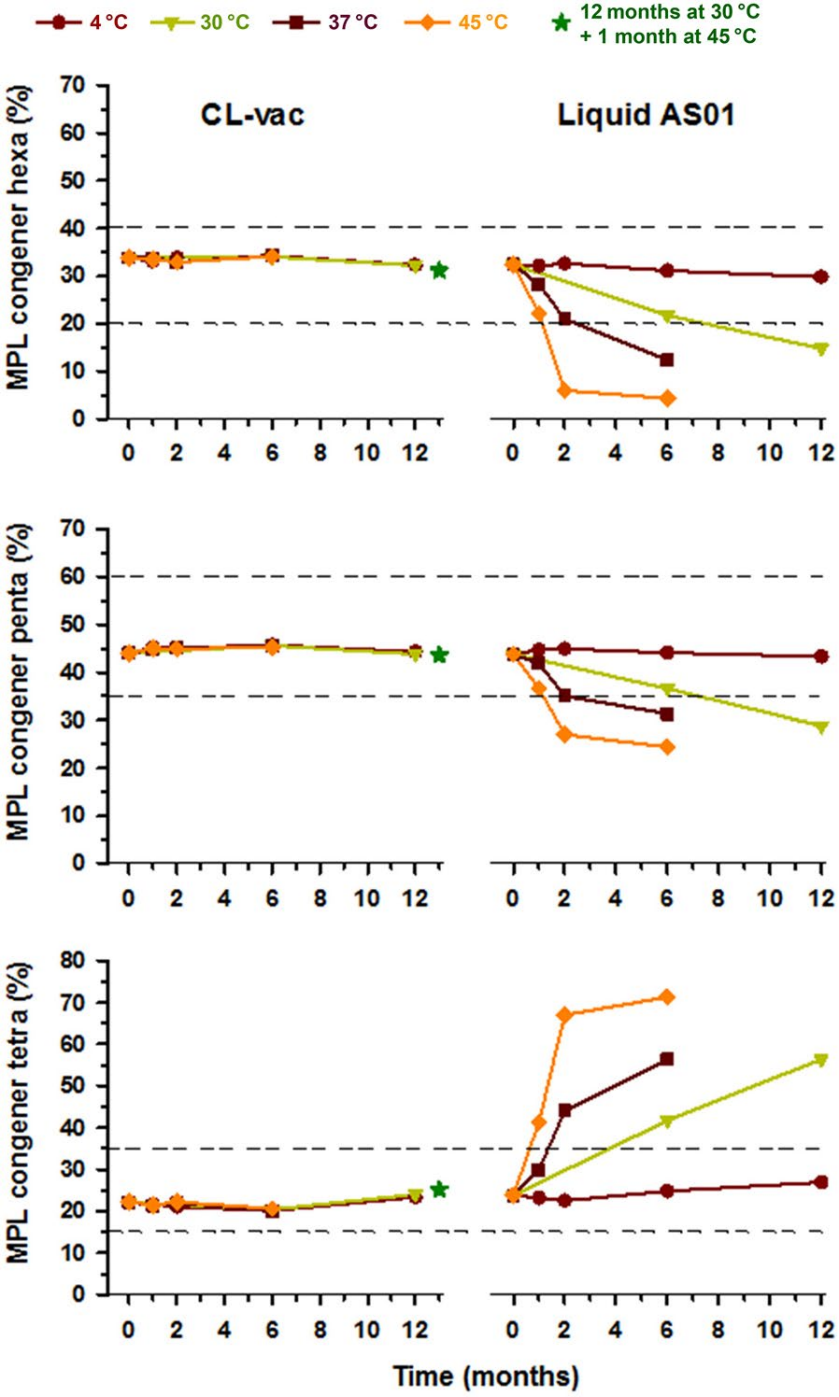
Evolution of MPL congeners proportions upon storage. Liquid AS01 and CL-vac were stored at 4, 30, 37 or 45  °C for up to 12 months and the proportions of MPL congeners were determined at different time points. A short heat expedition was made for CL-vac consisting of 1 month at 45  °C after storage for 12 months at 30  °C and the proportions of MPL congeners were also measured at this final time point. The dashed lines indicate the respective accepted congener proportions set for the release of the adjuvant in liquid form (between 15 and 35%, 35 and 60%, and 20 and 40%, for tetra, penta and hexa, respectively)

The integrity of MPL was evaluated by the proportion of its different congeners (Fig. 8). The tetra forms are expected to represent between 15 and 35% of the total MPL, whereas the penta form and the hexa forms are expected to represent between 35 and 60% and 20 and 40%, respectively. Liquid AS01 stored at 4  °C remained within these limits, even after 1 year. However, when stored at 30  °C, the proportion of the tetra congener in liquid AS01 increased progressively and exceeded the 35% limit after 4 months. Conjointly, the proportion of penta and hexa decreased. The phenomenon was faster at 37 and 45  °C. In contrast, the proportions of MPL congeners remained stable in CL-vac in all conditions of temperature and storage duration (Fig. 8).

### Visual inspection and electron microscopy analysis

The cake appearance of CL-vac after storage for 1 year at 30  °C or 6 months at 37  °C was similar to that of the product immediately after lyophilization. However, a slight shrinkage was observed in the cake after 6 months at 45  °C.

**Fig. 9:**
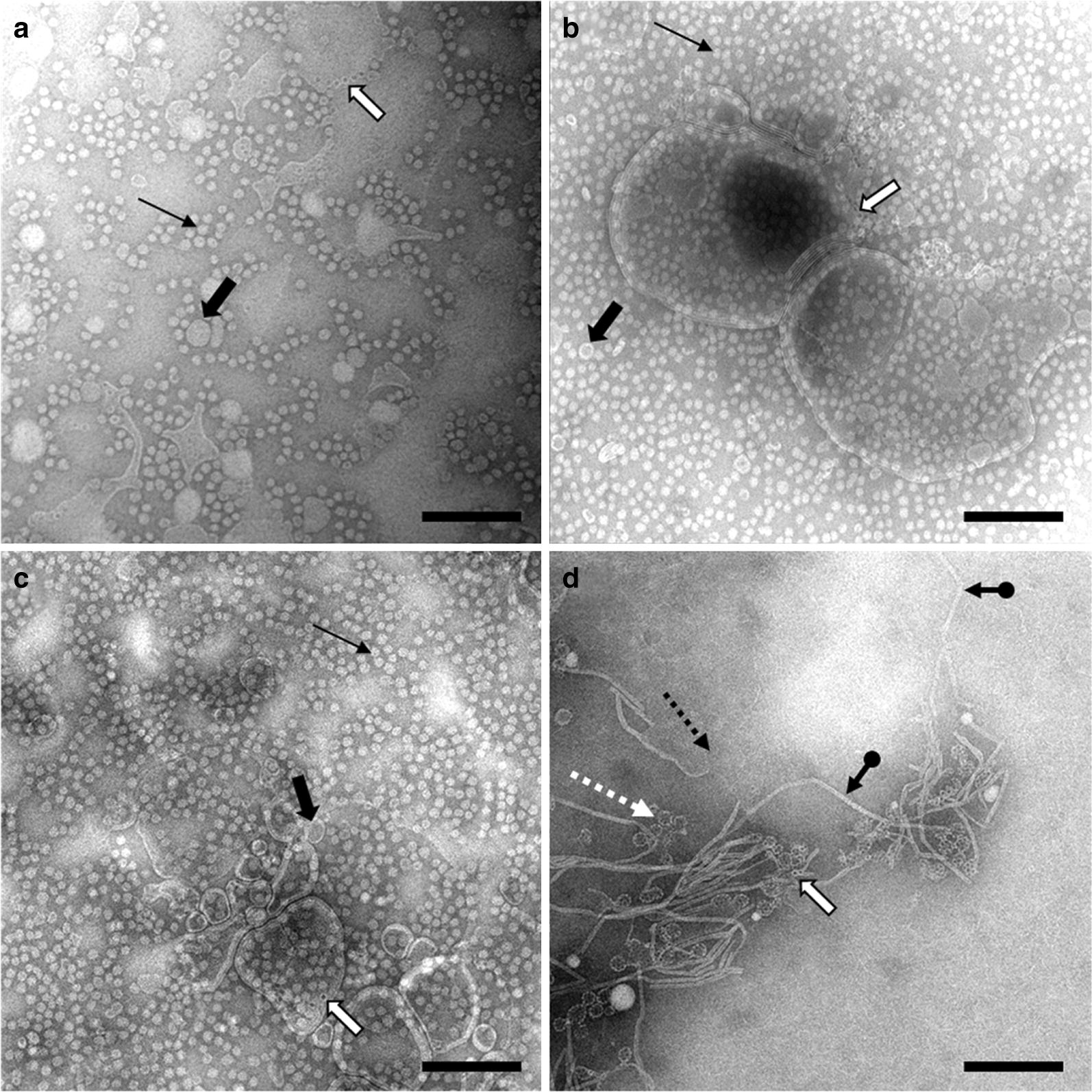
Electronic microscopy analysis of CL-vac after storage. Images obtained by TEM with negative staining of CL-vac after storage for 6 months at 37  °C (**a**), 6 months at 45  °C (**b**) or 1 year at 30  °C (**c**) before reconstitution, and liquid AS01 after storage for 6 months at 37  °C (**d**) are shown. White arrow, perforation of the liposome membrane. Thin black arrow, RTS,S particles. Thick black arrow, small liposome. Black dashed arrow, ghosts of liposome membrane. White dashed arrow, Iscom’s-like structures. Black dot-arrow, tubular structures

Electronic microscopy analysis showed no morphological alterations of RTS,S particles in CL-vac reconstituted after 6 months at 37  °C, 6 months at 45  °C, or 1 year of storage at 30  °C (Fig. 9a–c). Concerning AS01, all expected structures were present, such as small liposomes and liposomes with perforated membrane, which is indicative of a preserved AS01 pattern at all these time points. In contrast, electron microscopy analysis of AS01 alone kept 6 months at 45 °C in liquid form revealed a reorganization of the pattern (Fig. 9d), with many tubular and Iscom’s-like structures. Only rare liposomes or ghosts of liposomes with perforated membranes were still observed.

### Immunogenic properties

**Fig. 10:**
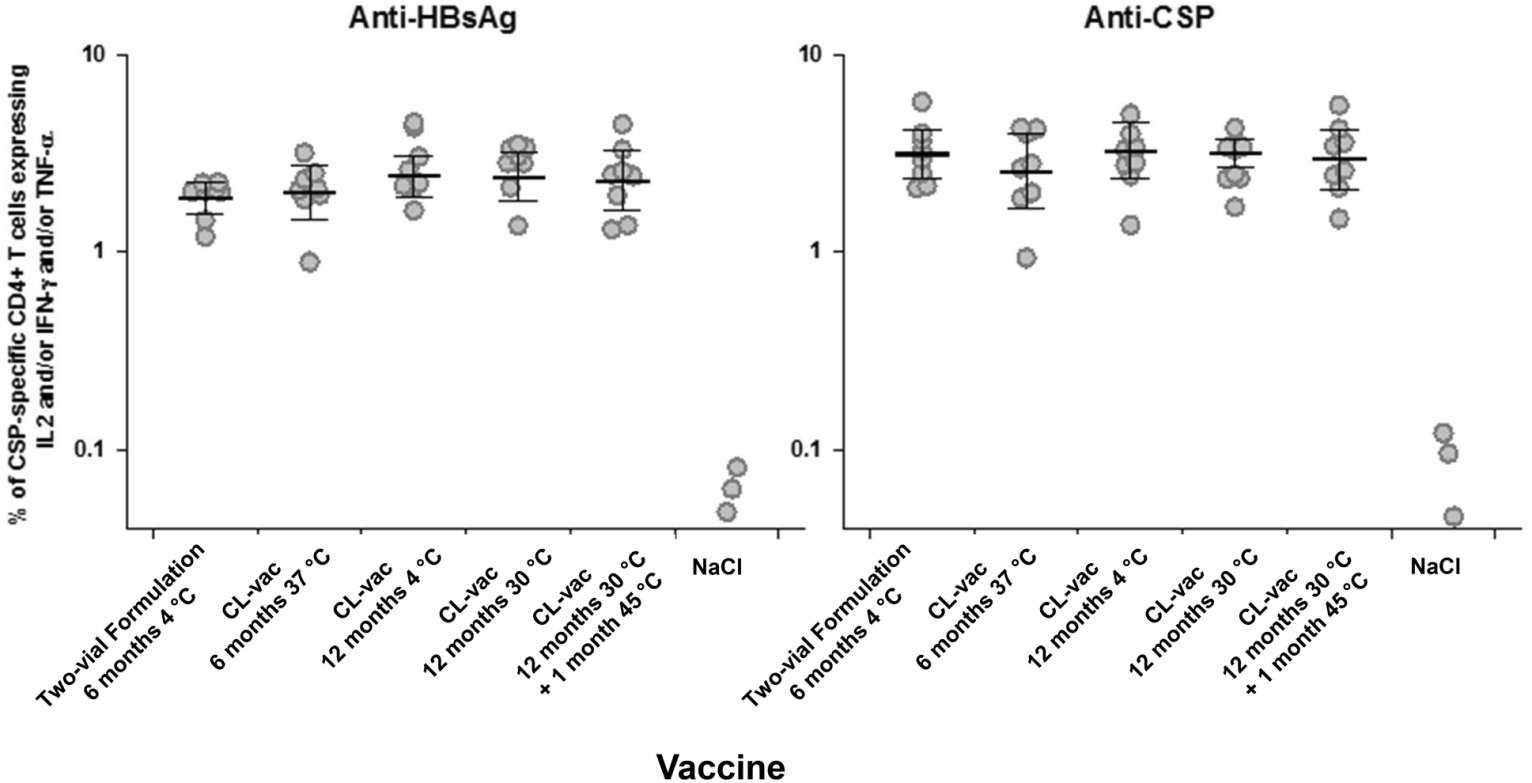
Evaluation of the immunogenic properties of CL-vac after storage. CL-vac was stored at 37  °C for 6 months or at 4 or 30  °C for up to 12 months. In addition, CL-vac was stored for 12 months at 30  °C followed by 1 month at 45  °C (heat excursion). After reconstitution of the vaccine, mice were immunized three times intramuscularly and the induced cellular responses were evaluated 7 days after the third immunization by intracellular cytokine staining, measuring the percentage of CSP- and HBsAg-specific CD4 + T cells expressing IFNγ and/or IL-2 and/or TNF. Immunization with the RTS,S/AS01 two-vial formulation was used as control. One dot represents one mouse. The black bars are geomeans with 95% CIs. The statistical analysis aimed to demonstrate equivalence between the reference (two-vial formulation) and the other stability conditions in a sequential manner, starting with CL-vac 6 months 37  °C, then CL-vac 12 months 4  °C, CL-vac 12 months 30  °C, and CL-vac 12 months 30 °C + 1 month 45  °C. Full equivalence was found for CSP- and HBsAg-specific cellular responses

Mice were immunized with CL-vac samples reconstituted after different conditions of storage and the induced cellular and humoral responses were evaluated. We measured similar T-cell responses after immunization with CL-vac stored for either 6 months at 37  °C, 1 year at 4  °C or 1 year at 30  °C. Indeed, similar levels of HBsAg-specific and CSP-specific CD4^+^ T cells were elicited than after injection of the two-vial formulation stored for 6 months at 4  °C (Fig. 10). The capacity to induce HBsAg-specific and CSP-specific CD4^+^ T-cell responses in mice was not impaired either after storage of CL-vac for 1 year at 30  °C followed by 1 month at 45  °C.

**Fig. 11:**
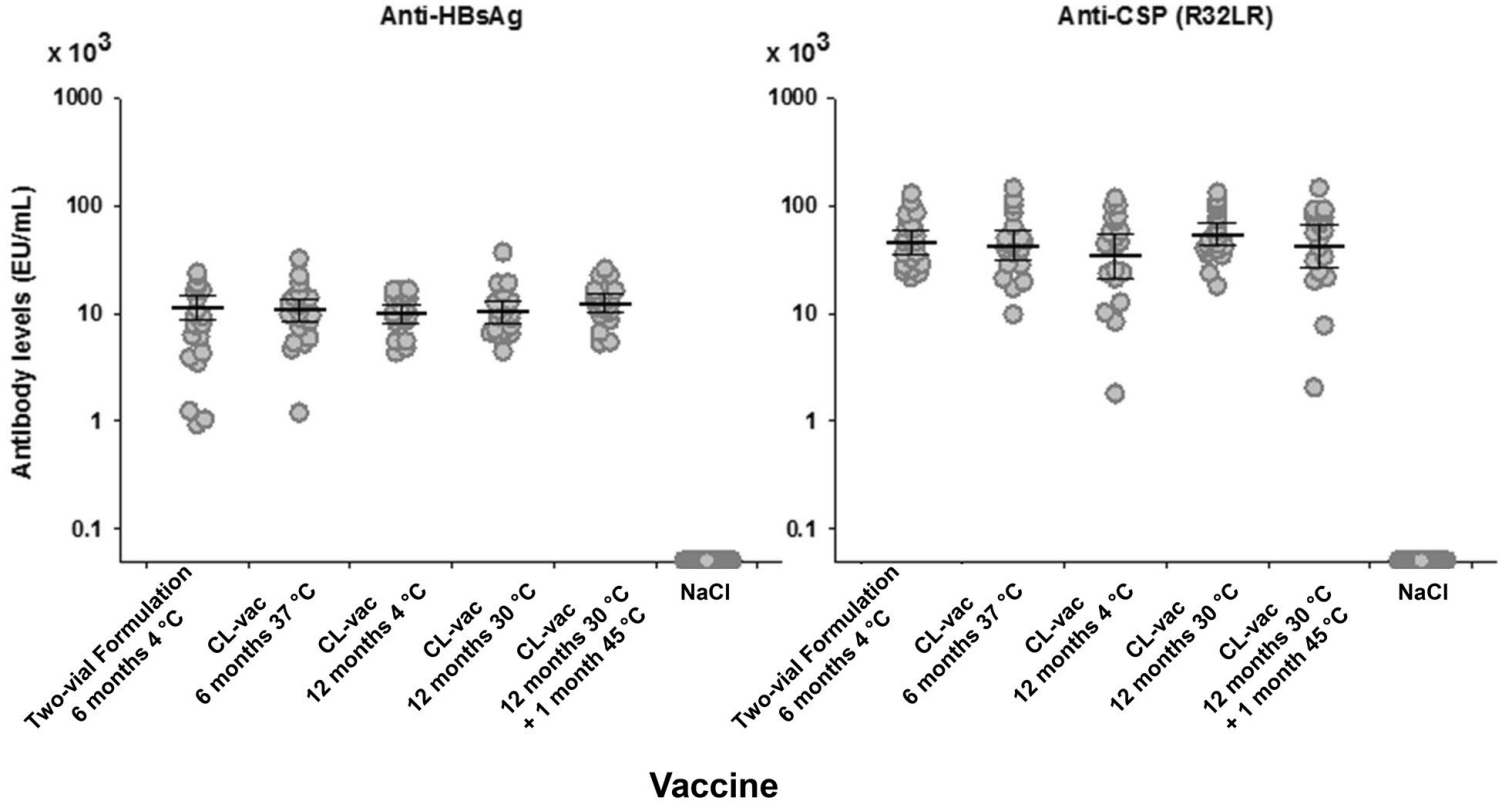
Evaluation of the immunogenic properties of CL-vac after storage. CL-vac was stored at 37  °C for 6 months or at 4 or 30  °C for up to 12 months. In addition, CL-vac was stored for 12 months at 30  °C followed by 1 month at 45  °C (heat excursion). After reconstitution of the vaccine, mice were immunized three times intramuscularly and the induced antibody responses were evaluated 2 weeks after the third immunization by ELISA. Immunization with the RTS,S/AS01 two-vial formulation was used as control. One dot represents one mouse. The black bars are geomeans with 95% CIs. The statistical analysis aimed to demonstrate equivalence between the reference (two-vial formulation) and the other stability conditions in a sequential manner, starting with CL-vac 6 months 37  °C, then CL-vac 12 months 4  °C, CL-vac 12 months 30  °C, and CL-vac 12 months 30  °C + 1 month 45  °C. Full equivalence was found for anti-HBsAg antibody responses. For the anti-CSP (R32LR) responses, equivalence to the two-vial formulation was found for CL-vac 6 months 37 °C, but not for CL-vac 12 months 4 °C, and consecutively not for the others (see “Results”)

Concerning the antibody response, the levels of anti-HBsAg antibodies in all groups were found equivalent to those obtained after immunization with the two-vial formulation stored for 6 months at 4  °C (Fig. 11). Concerning the levels of anti-CSP (R32LR), statistical equivalence could be shown for CL-vac stored for 6 months at 37  °C, but not for CL-vac stored for 1 year at 4  °C, and consecutively not for the rest of the groups, due to the sequential nature of the statistical analysis. However, a closer look at the results revealed that this outcome was principally due to the higher variability observed in the CL-vac-1-year-at-4  °C group. It is important to note that the comparison with the reference group lied just outside the lower limit of the 90% confidence interval, being 0.48. Also, when analysed in a non-sequential manner, the two other groups, 1 year at 30  °C and 1 year at 30  °C + 1 month at 45  °C, were found equivalent to the two-vial formulation (Fig. 11).

## Discussion

The malaria vaccine candidate RTS,S/AS01 is presented as a two-vial formulation, with the antigen in lyophilized form and the liposome-based Adjuvant System AS01 in liquid form. The lyophilized antigen needs to be reconstituted using the liquid AS01 adjuvant prior to administration of the vaccine. This two-vial presentation was the chosen approach at the time of early vaccine development to respond to technical constraints and deliver a high-quality vaccine. This formulation is stable for several years when kept refrigerated. However, the adjuvant part is less stable when exposed to higher temperatures.

The strategy to obtain a more thermostable malaria vaccine, here called CL-vac, was to co-lyophilize the RTS,S antigen together with AS01. In this procedure, sucrose was used as cryoprotectant and it is worth mentioning that the liposomes were formed before the addition of the cryoprotectant and not in its presence. The behaviour of these membranous structures in such conditions and of AS01 in general upon lyophilization was unknown at that moment and represented one of the main challenges of this study.

Overall, it was found that the co-lyophilization and reconstitution of CL-vac had only a minor or no impact on the physicochemical characteristics of the vaccine. RTS,S was not affected and MPL and QS-21, the two immunoenhancer components of AS01, showed similar properties as in non-lyophilized AS01. Only the liposome size appeared slightly different, which was attributed to the presence of polysorbate-80, as it is already present in the formulation before lyophilization of CL-vac. Liposomes that have incorporated polysorbate-80 have already been described as smaller than conventional liposomes [14], Once reconstituted, CL-vac was shown to be stable for 16 h at room temperature. From an immunological point of view, CL-vac and the two-vial formulation were found equivalent.

Further, as it was the purpose to obtain a malaria vaccine with increased thermostability, CL-vac was submitted to sustained exposure to temperatures that are otherwise detrimental to the two-vial formulation. The latter must be kept at 2 to 8  °C, according to its specifications, but it must be emphasized here that its sensitivity to higher temperatures is not due to the antigen. Indeed, RTS,S is lyophilized and we have again observed its excellent stability under this form in the present work. The necessity to store the two-vial formulation refrigerated is due to AS01 in liquid form. This feature is known and the present study confirms that the chemical integrity of AS01 in its liquid form is not maintained during prolonged storage at temperatures beyond 4  °C. In contrast, the chemical integrity of AS01 components upon storage was demonstrated in all CL-vac groups, which indicates the benefit of co-lyophilization. This is easily understandable, as QS-21 and MPL degradation is due to hydrolysis reactions that are slowed down after removal of water. The physico-chemical characteristics of CL-vac were unchanged after 1 year at 30  °C, 6 months at 37  °C or 45  °C. In addition, after 1 year at 30  °C, CL-vac could also withstand one additional month at 45  °C. Finally, immunization with stored CL-vac induced equivalent frequency of HBsAg- and CSP-specific CD4^+^ T cell responses as those induced by the original two-vial formulation. Likewise, no relevant impact of storage could be found on the anti-CSP antibody responses elicited by CL-vac, compared with the two-vial formulation.

## Conclusion

In conclusion, the present study shows that it is possible to co-lyophilize RTS,S and AS01, which would be the first pre-requisite to make a thermostable malaria vaccine. Importantly, the feasibility of freeze-drying AS01 liposomes was here demonstrated for the first time. Overall, CL-vac was found to be an efficient thermostable counterpart to the candidate RTS,S/AS01 in a two-vial formulation. Such thermostable vaccine would not necessitate cold chain constraint as it was shown to withstand temperatures higher than 2 to 8  °C for long periods of time, suitable for use in malaria-endemic countries.

## Data Availability

All data generated or analysed during this study are included in this published article.

## Abbreviations

AS01: Adjuvant System 01
CL-vac: Co-lyophilized vaccine
CSP: Circumsporozoite protein
DLS: Dynamic light scattering
DOPC: 1,2-dioleoyl-sn-phosphatidylcholine
EM: Electron microscopy
ELISA: Enzyme-linked immunosorbent assay
HBsAg: Hepatitis B surface antigen
HPLC-SEC: Size exclusion high performance liquid chromatography
MPL: 3-*O*-desacyl-4’-monophosphoryl lipid A
PBS: Phosphate-buffered saline
PDI: Polydispersity index
QS-21: *Quillaja saponaria* fraction 21
SDS-PAGE: Sodium dodecyl sulfate–polyacrylamide gel electrophoresis
ZAD: Z-average diameter
